# Suppression of Elastic Scattering of CH_4_ by Graphene Passivation of Ni(111)

**DOI:** 10.1021/acs.jpcc.4c06132

**Published:** 2024-11-26

**Authors:** Amjad Al Taleb, Francesc Viñes, Daniel Farías

**Affiliations:** † Departamento de Física de la Materia Condensada, 202533 Universidad Autónoma de Madrid, 28049 Madrid, Spain; ‡ Departament de Ciència de Materials i Química Física & Institut de Química Teòrica i Computacional (IQTCUB), 16724 Universitat de Barcelona, c/Martí i Franquès 1, 08028 Barcelona, Spain; § Instituto “Nicolás Cabrera”, 202533 Universidad Autónoma de Madrid, 28049 Madrid, Spain; ∥ Condensed Matter Physics Center (IFIMAC), 202533 Universidad Autónoma de Madrid, 28049 Madrid, Spain

## Abstract

We report high-resolution angular diffraction measurements of CH_4_ and Ne from Ni(111) and from a graphene (Gr) layer grown on Ni(111), whose effective atomic mass is enhanced by the strong Gr–Ni interaction. The incident energies are between 43 and 68 meV for Ne, and between 62 and 108 meV for CH_4_ beams. Sharp diffraction features are observed with Ne beams from both Ni(111) and Gr/Ni(111) surfaces. However, using methane beams, clear diffraction peaks are observed only from Ni(111), with broad angular distributions measured from Gr/Ni(111), as expected for classical particles, with no quantum features. This is surprising, since Ne and methane have similar masses and therefore a comparable quantum behavior is expected for the same incident energy. This effect is mainly due to the larger physisorption well of CH_4_ on Gr/Ni(111), as shown by DFT calculations, in addition to the larger corrugation of the potential energy surface and the excitation of phonon modes of the graphene overlayer.

## Graphical abstract

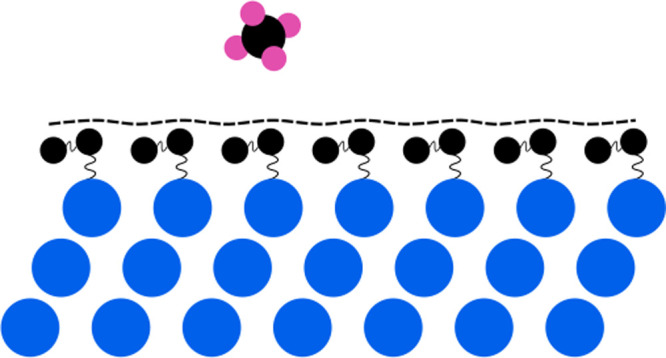

## Introduction

The dynamics of gas-surface interactions has been extensively studied using supersonic molecular beams, which allow their study by separately controlling the energy states of the gas species, the surface temperature, and the incident directions.^1,2^ Molecular beams with a variable energy can be used to bridge the pressure gap in ultrahigh vacuum conditions, as demonstrated recently in a quantitative way for the oxidation of Cu(111).^3^

The dissociative chemisorption of methane on transition-metal surfaces has been a target of surface science studies for decades due to its role as the rate-limiting step in the steam reforming reaction.^4–7^ In particular, several experimental studies have investigated the dependence of the dissociative chemisorption probability of CH_4_ as a function of the incident energy and surface temperature on both Ni(111)^8–14^ and Pt(111)^13–19^ surfaces. Since the scattering pattern directly reflects the gas-surface interaction, including elastic and inelastic scattering and trapping-desorption, the analysis of the scattering patterns has been carried out as one of the important approaches to understand the gas-surface interaction.

Surface diffraction is usually observed with atoms (He, Ne, Ar) and light molecules (H_2_/D_2_).^20–26^ When heavy molecules are used (N_2_, O_2_, CH_4_), a classical scattering distribution is observed.^27–29^ The case of methane is particularly interesting because its mass is very similar to that of Ne, i.e., their de Broglie wavelengths are comparable at the same kinetic energy. Thus, from the viewpoint of the experimental angular resolution required to see diffraction features, the problem is identical. However, diffraction from metal surfaces is routinely observed with Ne atoms, but not with CH_4_ molecules, which produce instead a broad angular distribution, as expected for classical particles. The only exception is a study reported by Andersson et al.^27^ who observed rotationally inelastic diffraction peaks in the scattering of CH_4_ from Cu(111) at 10 K, i.e., at temperatures where CH_4_ physisorbs, requiring continuous cleaning of the surface by pulsed laser heating during diffraction measurements.

We have recently shown that by choosing the appropriate experimental conditions, pure elastic diffraction of monochromatic CH_4_ beams can be observed in the scattering from Ni(111) and Ir(111) surfaces kept at 110 K, using incident energies in the range of 50–100 meV, i.e., well below the dissociation barrier.^30,31^ These results represented the first observation of surface diffraction with a large molecule above the desorption temperature of CH_4_ (ca. 45 K), and proved that quantum coherence is preserved, despite the small separation between CH_4_ rotational levels and the interaction with surface phonons. In particular, our studies show that the main reason for the absence of diffraction features with CH_4_ beams is given by the strong Debye–Waller attenuation, i.e., by the combination of a large value of the physisorption well *D* with a relatively low surface Debye temperature.

While our previous work^31^ focused mainly on CH_4_ scattering from clean Ni(111), here we present a comparative study of Ne and CH_4_ scattering from Ni(111) and from a graphene (Gr) passivated Ni(111) surface (Gr/Ni(111)). Gr forms a (1 × 1) structure on Ni(111)^32^ and, owing to the strong C–Ni bonding, the effective mass at the surface is enhanced. As expected, sharp diffraction features are observed with Ne beams from both Ni(111) and Gr/Ni(111) surfaces. However, using CH_4_ beams clear diffraction peaks are observed only from Ni(111), whereas broad angular distributions are measured from Gr/Ni(111). An analysis based on DFT calculations show that this is mainly due to the larger physisorption well of CH_4_ on Gr/Ni(111), in addition to the larger corrugation of the corresponding potential energy surface (PES). The role played by the Gr phonon modes in the scattering process is also discussed.

## Experimental Section

The experiments were performed with a high resolution molecular beam scattering time-of-flight (TOF) spectrometer, described in detail elsewhere.^33,34^ Essentially, the molecular beam, produced in a high pressure free jet expansion of the gas, is modulated by a rotating disk chopper for TOF measurements. The pure CH_4_ beam is formed by expanding the gas from a 20 μm diameter nozzle operated at 7 bar, while 15 bar were used for Ne with the same nozzle. The molecules scattered from the sample, after traveling trough three differentially pumped stages along the 1.7 m long drift tube, are detected by means of a mass sensitive detector. The angle between incident and scattered beam, in a planar geometry, is fixed at a total angle θ_SD_ = θ_i_ + θ_f_ = 105.4°. The angular distributions are measured by rotating the crystal by angle steps of Δθ_i_ = 0.018° around an axis perpendicular to a plane defined by the incoming beam and the normal to the sample surface. Throughout this paper we will use the term scattering angle which will mean the scattering angle relative to the specular position.

The beam energy has been varied by changing the nozzle temperature between 200 and 315 K. The corresponding changes of kinetic beam energies are between 43 and 68 meV for Ne and between 62 and 108 meV for CH_4_ beams. The different range of energies obtained for the same range of nozzle temperatures is a consequence of the different behavior of Ne and CH_4_ when the beam is formed at the nozzle. The kinetic energy of the CH_4_ beam has been determined by doing time-of-flight measurements using a dedicated detector installed in the direct beam path. We have performed such a calibration using this setup for nozzle temperatures in the range 200–400 K in a previous study.^30^ The corresponding energy spread (full with at half-maximum, fwhm) varies from 2.2 to 5% for Ne and 28.6 to 35% to for CH_4_. From measurements performed under similar expansion conditions, our CH_4_ beam population can be estimated to be 30% in *J* = 0, 50% in *J* = 1 and 20% in *J* = 2.^35^ Due to the low energies employed in our experiments (62–108 meV), molecular vibrations cannot be excited in the scattering process, and therefore only rotational transitions of CH_4_ molecules are observed in the scattering from the Ni(111) surface. The high angular resolution of this machine, combined with its large dynamical range, make it the ideal tool to resolve Rotationally Inelastic Diffraction (RID) peaks. The beam incident energy has been determined by performing time-of-flight measurements using a dedicated detector installed in the direct beam path.^30^

The Ni(111) crystal used in this study is a disk with a diameter of 8 mm and a thickness of 2 mm. The crystal was mounted on the sample holder which can be heated by electronic bombardment or cooled to 100 K using liquid nitrogen. The sample temperature was measured with a K-type thermocouple spot-welded to the sample edge. Clean Ni(111) surfaces were prepared in UHV by repeated cycles of ion sputtering and flash-annealing at ca. 1400 K. The Gr layer was prepared by exposure to ethylene at a pressure *P*_C_2_H_4__ = 5 × 10^–7^ mbar for 20 min while keeping the surface at 750 K. Further details on the sample preparation can be found elsewhere.^36–38^

The cleanliness and azimuthal alignment of the sample have been monitored by means of the analysis of He-diffraction angular distributions,^39^ as well as by low electron energy diffraction (LEED). The inertness of the Gr/Ni(111) surface was checked by monitoring the specular He-intensity while dosing oxygen from the background. Graphene-passivated samples exhibited a constant He-reflectivity, even 1 week after sample preparation when kept at a base pressure *p* = 5 × 10^–10^ mbar.

## Computational Details

The interaction strength of methane on Ni(111), Pt(111), and Gr/Ni(111) surfaces was estimated by Density Functional Theory (DFT) calculations, carried out using the Vienna Ab Initio Simulation Package (VASP).^40^ To this end, slab models were used, accounting for surface’s periodic boundary conditions, consisting of six metal layers and constructed from optimized bulks, where all interlayer distances were fully optimized.^41^ Bulk was previously described using the Perdew–Burke–Ernzerhof (PBE) exchange–correlation DFT functional,^42^ known to duly describe such systems^41^ plus a functional of choice when describing Gr/Ni(111).^43^ To reduce CH_4_ molecule interaction with replicated ones within periodic boundary conditions, *p*(3 × 3) supercells were employed for Ni(111) and Pt(111) surfaces, with an optimal **k**-points Γ-centered Monkhorst–Pack grid of 7 × 7 × 1 dimensions. During the surface and CH_4_ adsorption optimizations, a single CH_4_ was placed on one side of the slab, where both molecule and the three outermost layers were fully relaxed, whereas the bottommost three were fixed −3 + 3 approximation. The electronic structure convergence criterion was set to 10^–6^ eV, and structures were considered optimized when forces acting on atoms were below 0.01 eV Å^–1^. Notice that test calculations using larger number of **k**-points or basis set yielded variations on interaction strengths below 30 meV.

The slab models contained a vacuum region of 10 Å to avoid interaction between replicated slabs, and a plane-wave basis set for valence electrons up to a kinetic energy of 415 eV was employed, while core electrons were described by the Projector Augmented Waved (PAW) method.^44^ To account for dispersive forces during CH_4_ adsorption optimizations and for pristine surfaces optimization the PBE-based functional optPBE-vdW was employed,^45^ a functional including van der Waals (vdW) contribution, with an accuracy similar to other vdW functionals and dispersive forces corrections, as quantified on Gr/Ni(111) surface interaction,^46^ guaranteeing that results trends are functional-independent. As far as the Gr/Ni(111) system is concerned, the most stable top-fcc configuration has been modeled and used, as reported in the past.^43^ Even if a bridge-top situation is energetically accessible, one can assume a very similar CH_4_ adsorption strength. The methane molecule was placed over high-symmetry sites on each employed model (see Figure 1) and initially placed 3 Å above the surface, with either one, two, or three H atoms pointed toward the surface, including two possible orientations over the surface.

**Figure 1:**
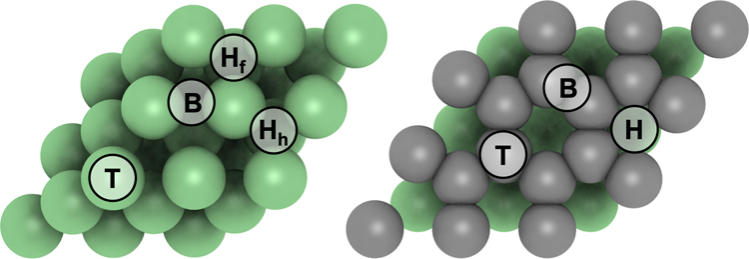
Top views of the Ni(111) (left) and Gr/Ni(111) (right) models, with the sampled adsorption sites, including top (T), bridge (B), and hollow site in case of Gr/Ni(111) (H), and two different 3-fold hollow sites on Ni(111); the hollow fcc, Hf, with a metal atom of the subsurface layer directly underneath, and the hollow hcp, with a metal atom of the second subsurface layer directly underneath. Ni and C atoms are shown as green and gray spheres, respectively, with shade increasing when going subsurface.

The interaction strength or adsorption energy, *E*_ads_, has been seized as

$$E_{\mathrm{ads}}=E_{{\mathrm{CH}}_{4}/M}-E_{{\mathrm{CH}}_{4}}-E_{M}$$ 1

where *E*_CH_4_/M_ is the total energy of the surface model with the adsorbed CH_4_, *E*_CH_4__ the total energy of CH_4_ molecule optimized Γ-point in a cubic box of 10 × 10 × 10 Å^3^ dimensions, and *E*_M_ the energy of the pristine surface model. Within this definition, favorable adsorption energies are defined negative.

## Results

Considering only elastic scattering (i.e., ignoring energy transfer between molecule and surface), we can say that when a molecule impinges on a surface it can either scatter elastically giving diffraction peaks in the angular distribution of the scattered beam or, under certain incident conditions, energy exchange can take place between internal degrees of freedom of the molecule resulting in RID peaks. In this process, the incident molecules convert part of their translational energy into excitation of a rotational quantum level when colliding with the surface. RID peaks show up in the form of additional diffraction peaks in the angular distributions, and are usually observed with H_2_ and D_2_ beams.^47^

The Gr/Ni(111) system is a benchmark example of a strongly interacting system. Owing to a misfit of less than 2%, Gr forms a (1 × 1) structure on Ni(111).^32^ The Gr (1 × 1) structure is formed by one C atom sitting on top of a Ni atom, whereas the second C atom occupies a hollow site.^32^ Figure 2 shows a comparison of the angular distributions of the scattering of CH_4_ from Ni(111) and Gr/Ni(111) measured at 110 K and at three different beam energies, indicated on each panel. In the scattering from Ni(111) (pink curves), the most intense feature is the specular peak which appears at Δθ_f_ = 0°. Several RID peaks are observed, some of them with high intensity. The most intense RID peaks are the ones labeled as (00):12 and (00):03, which appear at positive angles with respect to the specular peak.^31^ Their intensities amount ca. 50% of the specular peak’s intensity. For comparison, the most intense RID peak observed in D_2_ scattering from NiAl(110) is about 10% of the specular intensity.^34^

**Figure 2:**
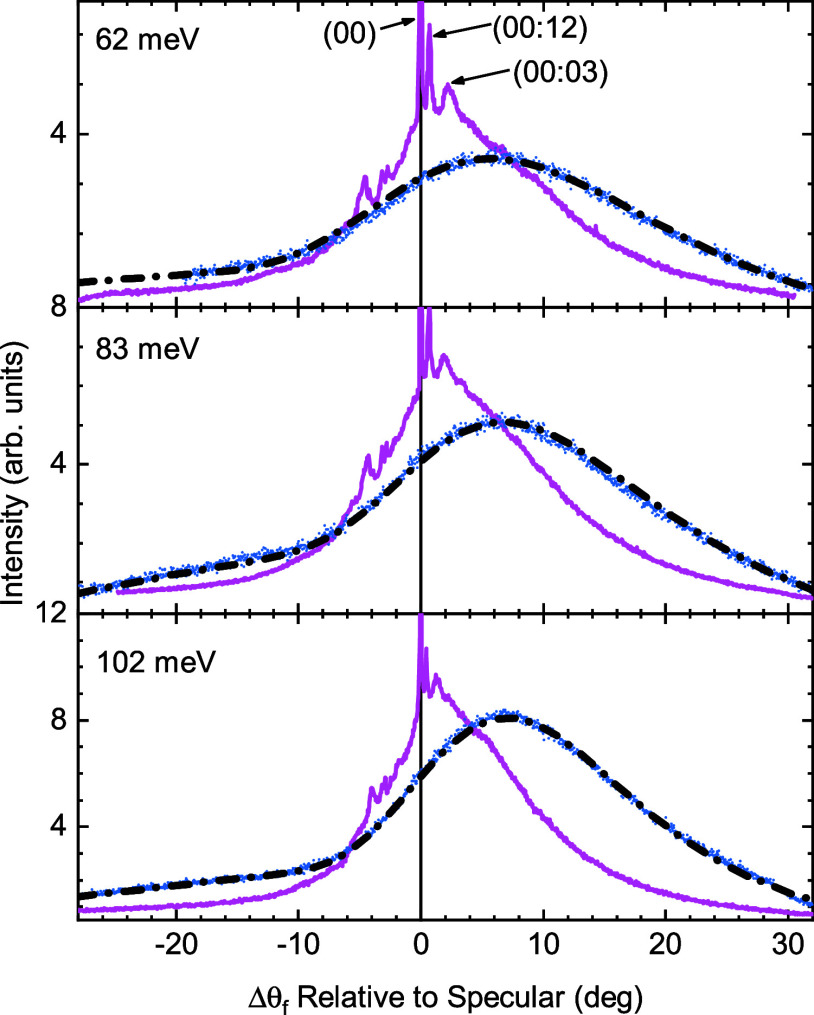
Angular distributions of CH_4_ beams scattered from Ni(111) (pink) and Gr/Ni(111) (blue) surfaces along the $\overset{®}{\Gamma M}$ high-symmetry direction of Ni(111) at various incident beam energies. The surface temperature is *T*_S_ = 110 K. The vertical black line indicates the specular reflection angular position. The black dashed curves were produced by adding the normalized angular distribution calculated using the washboard model to the background extracted from the experimental data (see text).

In addition, scattering from Ni(111) exhibits a wide inelastic scattering background centered around a scattering angle of ca. 3–5°. Moreover, there is a pronounced asymmetry in the intensities distribution with respect to the specular peak, which leads to higher intensities at 10° than at −10°. As pointed out in a recent theoretical study by Jackson,^48^ this could be due to increased trapping of back-scattered molecules. The normal component of the collision energy at 10° is 47 meV, which is reduced to 18 meV at −10°. In general, it means that for a fixed scattering geometry like the one used in our experiments, the magnitude of the back-scattered peaks is lowered relative to the forward–scattered peaks due to the increased trapping.

In contrast, RID peaks are absent in CH_4_ scattering from Gr/Ni(111) at all tested energies (blue curves). A broad distribution with no sharp features is observed, with a maximum shifted toward larger scattering angles (7°) which correspond to smaller angles of incidence, resulting in a larger perpendicular component of the incident beam energy. So the scattering intensity increases with increasing efficiency of perpendicular energy transfer. We can observe a small increase in the scattered intensities around −22°, which can be described using a cosine distribution that results from trapping-desorption events. A recent study based on the washboard^49^ model has shown that such a pronounced shift is expected for large values of the well depth *D*.^50^ As we show below, our simulations suggest that this is the case for CH_4_ scattering from Gr/Ni(111). The dashed black curves show the normalized results of this simulation, more details on the fit parameters later, after adding the trapping-desorption feature which was extracted from the corresponding experimental curves.

Figure 3 shows a similar comparison using a Ne projectile, where the intensity is plotted on a logarithmic scale. Ne sees a very smooth Ni(111) surface, leading to the appearance of first order diffraction peaks with low intensity. In contrast, Ne samples a more corrugated Gr/Ni(111) surface which results in more pronounced diffraction peaks. The intensity of the first order diffraction peak measured with Ne scattering from Ni(111) is 0.72% of the specular peak intensity. In comparison, the intensities of the (00):12 and (00):03 peaks in the 86.2 meV CH_4_ diffraction spectrum in Figure 2 are 53 and 47% of the specular peak, respectively. The corrugation amplitude seen by Ne from Gr/Ni(111) is estimated in 0.12 Å, to be compared with 0.07 Å as seen with HAS.^38^

**Figure 3:**
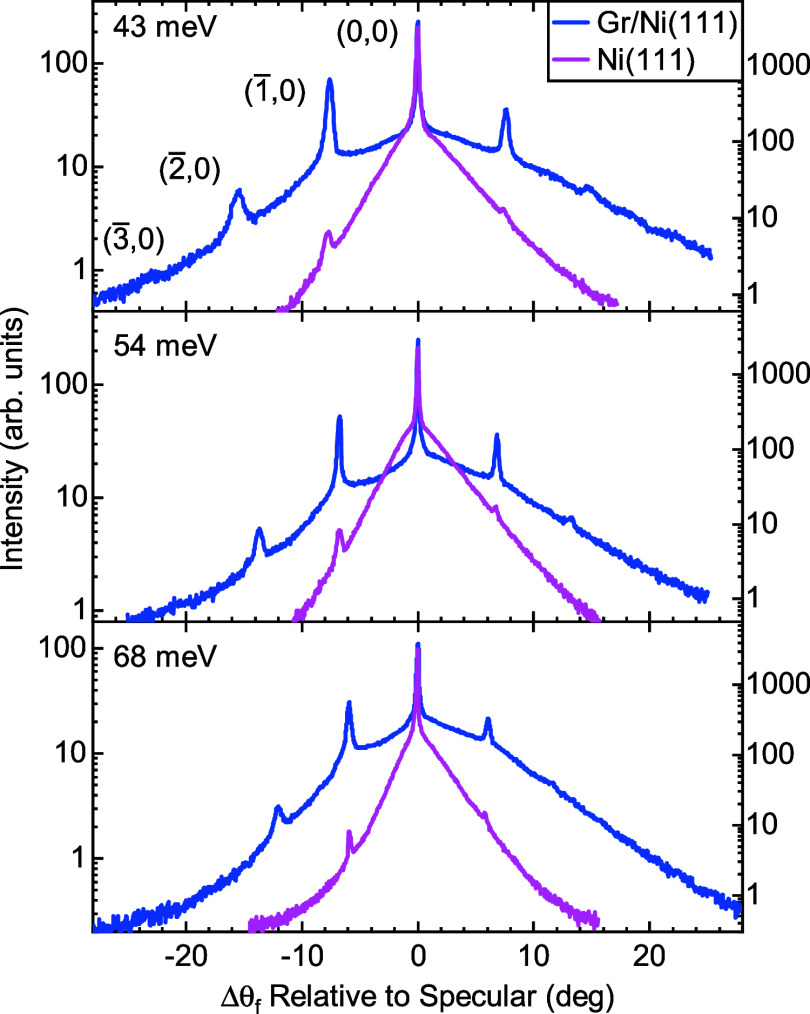
Angular distributions of Ne beams scattered from Ni(111) (pink) and Gr/Ni(111) (blue) surfaces along the $\overset{®}{\Gamma M}$ high-symmetry direction of Ni(111) at various incident beam energies. The surface temperature is *T*_S_ = 110 K. Selected diffraction peaks are annotated. Intensities are plotted on a logarithmic scale.

He scattering measurements from Ni(111) and Gr/Ni(111) show a larger Debye–Waller factor for the latter,^38^ a similar trend is expected for Ne scattering, and therefore a higher intensity of the inelastic scattering background from the graphene-covered surface for similar scattering conditions. We can see this behavior clearly in Figure 3 where the inelastic background in the scattering of Ne from Gr/Ni(111) is larger that that from Ni(111), the background shifts toward larger scattering angles (corresponding to smaller angles of incidence) with increasing energy of the incident beam.

Figure 4 compares the angular diffraction spectra measured with Ne and CH_4_ beams from Gr/Ni(111) at nearly the same incident energy and surface temperature (*E*_i_ = 68 meV for Ne and 62 meV for CH_4_). Ne intensity values are shown on the left vertical axis and those taken with CH_4_ beams on the right one. As mentioned above, the relatively high corrugation of Gr/Ni(111) leads to the appearance of high diffraction intensities in the case of Ne, where even second order peaks are detected. Intensities of first and second order diffraction peaks with respect to the specular one are 53 and 5%, respectively. It is obvious from this figure that CH_4_ scattering from Gr/Ni(111) is fundamentally different, since diffraction features are absent and just a broad angular distribution is observed, with its maximum shifted to Δθ_i_ ≈ 6°. Since CH_4_ and Ne have comparable masses, the Ne data prove that the origin of the different behavior observed for CH_4_ scattering cannot stem from the high incident mass, and should instead be related to differences in the van der Waals physisorption wells on the two surfaces investigated, as discussed in more detail below. The differences we observe in diffraction resemble the dramatic differences recently reported in the vibrational state distribution in state-to-state CH_4_ scattering experiments on Ni(111) and Gr/Ni(111).^51,52^

**Figure 4:**
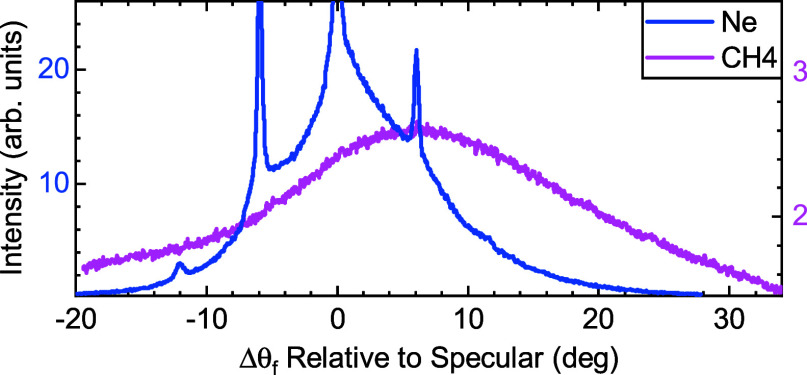
Angular distributions of Ne (blue) and CH_4_ (pink) scattering from Gr/Ni(111) under similar incident conditions, *E*_i_ = 68 meV for Ne and 62 meV for CH_4_. The surface temperature is *T*_S_ = 110 K.

From DFT calculations, after sampling the surface models with adsorbed CH_4_, the most stable sites were found to be η^3^ configurations, i.e., with three of its H atoms pointing toward the surface, with least stable η^1^ configuration, i.e., with only one H atom pointing toward the surface, being 24 meV less stable for Ni(111), and 33 and 49 meV for Pt(111) and Gr/Ni(111) models. In the most stable configurations, three C–H bonds are oriented toward the surface. The computed *E*_ads_ value is 200 meV for Ni(111) for methane sitting on a hollow fcc site, being the lowest adsorption energy of the three surfaces under study. A much larger interaction energy of 458 meV was obtained on Pt(111), with CH_4_ sitting on a hollow fcc site, which is consistent with the difficulty in obtaining any methane diffraction from this surface. Finally, on Gr/Ni(111), the computed well depth is of 275 meV, but now for CH_4_ adsorbed on a top site. This value is in between those of Ni(111) and Pt(111), and larger than the one obtained on Ni(111) by 75 meV. In the case of Ne interacting with Gr/Ni(111), it lays on the graphene hollows with a well depth of 53 meV.

Note that the present optPBE-vdW values may be larger than the derived experimental values, and so, the method seems to overestimate the interaction strengths, by at least 65 meV if one compares the computed values on Ni(111). However, the differences among the three surfaces studies are large enough to ensure that the trend would be kept if some other functional flavor were used.

Figure 5 shows on the top panel the experimental angular distribution of the scattered intensities of CH_4_ from Gr/Ni(111) at three different incident energies after removing the background and the trapping-desorption feature. On the bottom panel we show the calculated angular distribution of scattered intensity of CH_4_ using the washboard model^49^ discussed in details by Kondo et al.^50,53^ We used the attractive potential well depth *D* = 275 meV obtained from our DFT calculations for Gr/Ni(111) and surface temperature *T*_S_ = 110 K. The best fit was obtained using a surface atomic mass *M*_S_ = 322 amu and a surface corrugation parameter α = 1.4°, which gives a corrugation amplitude 0.01 Å using a surface periodicity of 2.46 Å. The larger surface effective mass and smaller corrugation measured by CH_4_ scattering compared to HAS^38^ could be primarily due to the larger size of the impinging molecule. Note that this value of surface corrugation corresponds to a simplified model based on simple classical binary collisions, whereas the actual high-dimensional PES is expected to exhibit a higher corrugation depending on the orientation of the incident CH_4_ molecule, as demonstrated recently by calculations performed for the CH_4_–Ir­(111) system, where the corrugation amplitude was found to be 0.20 Å.^30^

**Figure 5:**
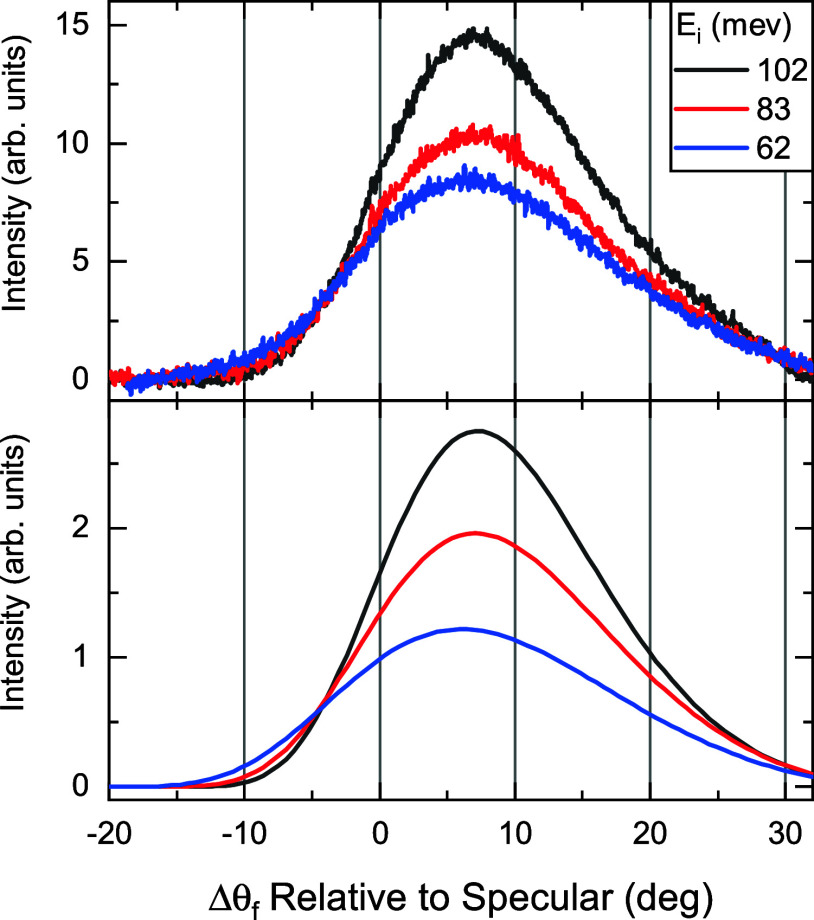
Top: angular distribution of CH_4_ from Gr/Ni(111) measured at various incident beam energies, after removing the trapping-desorption scattering data. The surface temperature is *T*_S_ = 110 K. Bottom: washboard model calculations for the same beam energies.

## Discussion

A useful tool to analyze the scattering of Ne and CH_4_ from solid surfaces is the Debye–Waller model. This model provides an easy way to take into account the inelastic scattering of the incoming atoms or molecules. The main observable effect of this is the exponential attenuation with surface temperature of the specular and diffraction peaks. This model relates relates the intensity *I*(*T*) of an elastic scattered peak with the intensity *I*_0_ from a lattice at rest by^54,55^

$$I(T)=I_{0}e^{-2W(T)}$$ 2

where exp­[−2*W*(*T*)] is the Debye–Waller factor. This expression has been shown to describe many He,^39^ H_2_,^26^ Ne, and Ar diffraction experiments rather well.^56–58^ For the scattering of thermal atoms from surfaces, the effect of the attractive well near the surface is usually taken into consideration by including the so-called “Beeby correction”.^59^ For the specular beam, *W*(*T*) can be easily expressed as a function of the incident beam energy *E*_i_ and the angle of incidence θ_i_:

$$W(T)=\frac{12m(E_{i}\cos^{2}\;\theta_{i}+D)T}{Mk_{B}\Theta_{D}^{2}}$$ 3

where *M* is the mass of a surface atom, *m* is the mass of the incoming particle, *k*_B_ is the Boltzmann constant, Θ_D_ is the surface Debye temperature, and *D* is the potential well depth. Eq 3 indicates that the strength of the diffraction intensities should be highest for grazing incidence, low incidence energy and low surface temperatures, in agreement with experiment.^39^

A recent comparison of the temperature dependence of Ne and CH_4_ specular and RID peaks measured from Ir(111) has shown that the Debye–Waller model also describes well the coherent scattering of CH_4_ molecules.^30^ A major conclusion of that study is that for CH_4_ beams a reduction of absolute specular intensity by a factor 25 with respect to Ne diffraction is expected. This strong reduction is mainly due to the large value of the well depth (*D*_CH_4__ ≈ 135 meV for Ni(111)^60,61^ ) as compared to typical values for Ne–metal interactions (*D*_Ne_ ≈ 15 meV). Since specular Ne diffraction is usually limited to 10–20% of the incoming beam intensity,^39^ the absolute specular intensity measured with CH_4_ beams will be typically at ca. 1%, just as found experimentally.

To see how this argument applies to a real case, it is useful to compare angular distributions of CH_4_ from Ni(111) and Pt(111) measured under identical incident conditions (see Figure 6). As mentioned above, the spectrum from Ni(111) shows diffraction features in the form of sharp RID peaks, whereas that from Pt(111) exhibits a broad angular distribution, with its maximum shifted to ca. 2.5°. This broad angular distribution is quite similar to the one observed from Gr/Ni(111) (shown in Figure 5). The values of *M*Θ_D_^2^ are very similar on both surfaces (*M*Θ_D_^2^ = 16.1 × 10^6^ amu K^2^ and *M*Θ_D_^2^ = 16.5 × 10^6^ amu K^2^ for Ni(111) and Pt(111), respectively).^30,62^ According to the Debye–Waller model (eqs 2 and 3), this means that CH_4_ scattering intensity from both surfaces is expected to be quite similar. Thus, the data in Figure 6 suggest that the potential well of CH_4_ interacting with Pt(111) is much larger than the well-depth of the CH_4_ -Ni(111) interaction. This is actually the case, the corresponding values estimated from experiment for Ni(111)^60,61^ and Pt(111)^63,64^ are *D* = 135 meV and *D* = 160 meV, respectively. The trend of these values are in line with the trend observed from DFT calculations, with *D* = 200 meV for Ni(111) and *D* = 458 meV for Pt(111). In the case of Ne on Gr/Ni(111), the value of 53 meV is comparable to the aforementioned values of Ne on metal surfaces. Although the absolute values are overestimated, the trend should be correct.

**Figure 6:**
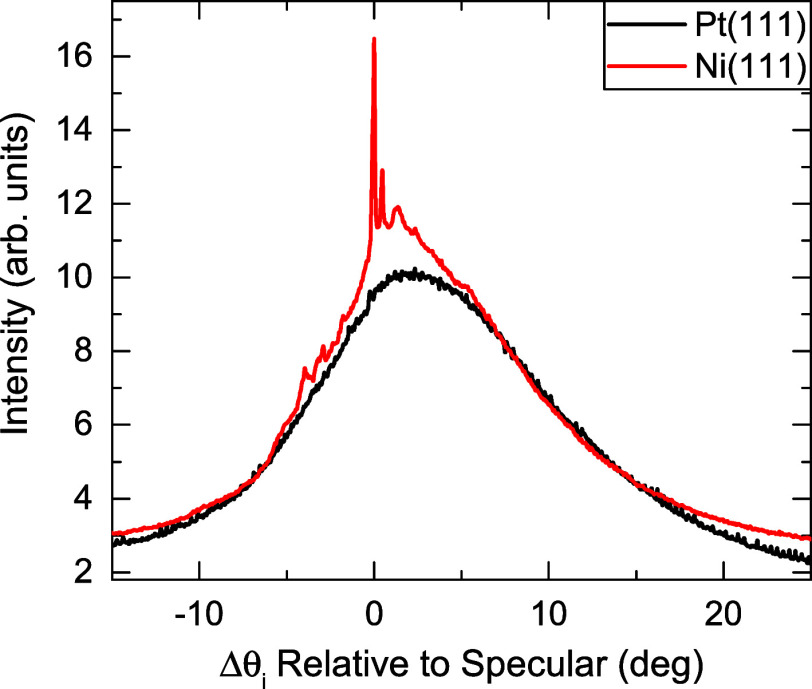
Angular distributions of CH_4_ scattering from Ni(111) (red) and Pt(111) (black) under identical incident conditions, *E*_i_ ≈ 102 meV and *T*_S_ = 110 K.

The well depth obtained from the calculations for Gr/Ni(111) is *D* = 275 meV, larger than the one obtained for Ni(111). The larger *D* value obtained for Gr/Ni(111) suggests that even lower intensities are expected for CH_4_ scattering as compared to scattering from Ni(111). In addition, due to its larger corrugation amplitude, more diffraction and RID channels will be opened on Gr/Ni(111), leading to a general decrease of coherent intensities.

Finally, on Gr/Ni(111), an additional dissipation channel to be considered is phonon excitation of the Gr layer. The phonon dispersion of Gr/Ni(111) has been measured by HREELS along both the $\overset{®}{\Gamma K}$ ^37^ and the $\overset{®}{\Gamma M}$ direction.^65^ The most relevant features observed are the softening of the LO, TO and ZO modes. The acoustic phonons look similar, with the presence of essentially the same Rayleigh wave on both clean Ni(111) and Gr/Ni(111) surfaces.^38,66^ In addition, the ZA branch does not go to zero for Δ*K* → 0 due to the symmetry breaking induced by the Gr–Ni interplanar force constants. As a consequence, on Gr/Ni(111), the flexural ZA mode has an energy of ∼20 meV at Γ̅, which provides an additional inelastic channel not available on Ni(111).

Perhaps the most important result for understanding our data is the softening of the ZO mode in Gr/Ni(111). The dispersion curve of this mode goes from 90 meV at Γ̅ to ca. 60 meV at the *K̅* and *M̅* points. This provides a new dissipation channel at the incident energies used in our experiments. It can be argued that excitation of this mode at the relatively low incident energy of 62 meV is unlikely, especially considering that the normal component of the collision energy is roughly half of this value. However, we have to keep in mind that, due to Beeby correction, the value of the potential well depth *D* should be added to the perpendicular energy component. The result is a value of about 150 meV for the normal component of the collision energy for CH_4_, which should be sufficient to excite optical phonon modes in Gr/Ni(111).

## Conclusions

In summary, we have presented a comparative study of Ne and CH_4_ scattering from Ni(111) and Gr/Ni(111) surfaces at incident energies between 43 and 108 meV. Whereas clear diffraction features are observed with Ne beams from both Ni(111) and Gr/Ni(111), sharp diffraction peaks are observed only from Ni(111) using CH_4_ beams, whereby broad angular distributions are measured from Gr/Ni(111). This surprising behavior is mainly due to the larger physisorption well *D* of CH_4_ on Gr/Ni(111) as compared to Ni(111), as shown by DFT calculations, together with a general decrease of elastic scattered intensity due the larger corrugation of the Gr/Ni(111) PES. Finally, a larger *D* leads to a larger perpendicular energy component of CH_4_ molecules, making excitation of soft graphene modes easier than with Ne atoms.
